# Efficacy and safety of olopatadine-mometasone combination nasal spray for the treatment of seasonal allergic rhinitis^☆^

**DOI:** 10.1016/j.waojou.2026.101341

**Published:** 2026-02-13

**Authors:** Yutong Sima, Xueyan Wang, Tao Zhang, Zhiwei Cao, Wei Chen, Fang Quan, Xiaoyong Ren, Yi Yang, Shiping Bao, Lifeng Xie, Changqing Zhao, Qinna Zhang, Zhimin Xing, Huifang Zhou, Jianjun Chen, Qingquan Hua, Ling Zhou, Xiaobing Zhang, Xiong Chen, Chao Li, Ruixia Ma, Hua Zhang, Zhendong Xu, Mei Han, Xiangdong Wang, Luo Zhang

**Affiliations:** a Department of Otorhinolaryngology Head and Neck Surgery, Beijing Tongren Hospital, Capital Medical University, Beijing, China; b Beijing Laboratory of Allergic Diseases, Beijing Municipal Education Commission and Beijing Key Laboratory of New Medicine and Diagnostic Technology Research for Nasal Disease, Beijing Institute of Otolaryngology, Beijing, China; c Allergy Centre, Beijing Shijitan Hospital, Capital Medical University, Beijing, China; d Department of Otorhinolaryngology Head and Neck Surgery, Tianjin Union Medical Center, The First Affiliated Hospital of Nankai University, Tianjin, China; e Department of Otorhinolaryngology Head and Neck Surgery, Shengjing Hospital of China Medical University, Shenyang, Liaoning Province, China; f Department of Otorhinolaryngology, Central Hospital of Wuhan, Tongji Medical College, Huazhong University of Science and Technology, Wuhan, Hubei Province, China; g Department of Otorhinolaryngology Head and Neck Surgery, The First Affiliated Hospital of Xi'an Jiaotong University, Xi'an, Shaanxi Province, China; h Department of Otorhinolaryngology Head and Neck Surgery, The Second Affiliated Hospital of Xi'an Jiaotong University, Xi'an, Shaanxi Province, China; i Department of Otorhinolaryngology, Beijing Hospital, National Center of Gerontology, Institute of Geriatric Medicine, Chinese Academy of Medical Sciences, China; j Department of Otorhinolaryngology Head and Neck Surgery, Beijing Youan Hospital, Capital Medical University, Beijing, China; k Department of Otorhinolaryngology Head and Neck Surgery, Peking University Third Hospital, Beijing, China; l Department of Otorhinolaryngology Head and Neck Surgery, Second Hospital of Shanxi Medical University, Taiyuan, Shanxi Province, China; m Department of Otorhinolaryngology Head and Neck Surgery, First Hospital of Shanxi Medical University, Taiyuan, Shanxi Province, China; n Department of Otolaryngology Head and Neck Surgery, Peking University People's Hospital, Beijing, China; o Department of Otolaryngology Head Neck Surgery, Tianjin Medical University General Hospital, Tianjin, China; p Department of Otorhinolaryngology, Union Hospital, Tongji Medical College, Huazhong University of Science and Technology, Wuhan, Hubei Province, China; q Department of Otorhinolaryngology Head and Neck Surgery, Renmin Hospital of Wuhan University, Wuhan, Hubei Province, China; r Department of Otorhinolaryngology, The First Affiliated Hospital of Heilongjiang University of Chinese Medicine, Haerbin, Heilongjiang Province, China; s Department of Otorhinolaryngology Head and Neck Surgery, The First Hospital of Lanzhou University, Lanzhou, Gansu Province, China; t Department of Otorhinolaryngology Head and Neck Surgery, Zhongnan Hospital of Wuhan University, Wuhan, Hubei Province, China; u Department of Otorhinolaryngology, The Second Hospital of Tianjin Medical University, Tianjin, China; v Department of Otorhinolaryngology Head and Neck Surgery, The First People's Hospital of Yinchuan, Yinchuan, Ningxia Hui Autonomous Region, China; w Department of Otolaryngology, The First Hospital of Xinjiang Medical University, Urumqi, Xinjiang Uygur Autonomous Region, China; x Department of Otolaryngology, Baotou Central Hospital, Baotou, Inner Mongolia Autonomous Region, China; y Department of Otolaryngology, The Affiliated Hospital of Changchun University of Chinese Medicine, Changchun, Jilin Province, China; z Department of Allergy, Beijing Tongren Hospital, Capital Medical University, Beijing, China; aa Research Unit of Diagnosis and Treatment of Chronic Nasal Diseases, Chinese Academy of Medical Sciences, Beijing, China

**Keywords:** Seasonal allergic rhinitis, GSP301, Olopatadine hydrochloride, Mometasone furoate

## Abstract

**Background:**

Patients with moderate-to-severe allergic rhinitis (AR) often experience a heavy clinical burden and require more medications to alleviate nasal symptoms. The aim of this study was to evaluate the efficacy and safety of GSP301, a fixed-dose combination nasal spray containing olopatadine hydrochloride and mometasone furoate, in patients with seasonal AR (SAR).

**Methods:**

In this multicenter, randomized, double-blind, parallel-group study, moderate-to-severe SAR patients were assigned at a 1:1:1 ratio to receive intranasal GSP301, olopatadine hydrochloride (OLO), or mometasone furoate (MF) for 14 days. The primary endpoint was the change from baseline in the average A.M. and P.M. 12-hour reflective total nasal symptom score (rTNSS). Secondary endpoints included changes in the instantaneous TNSS (iTNSS), individual nasal symptoms, reflective total ocular symptom score (rTOSS), instantaneous total ocular symptom score (iTOSS), individual ocular symptoms, and rhinoconjunctivitis quality-of-life questionnaire (RQLQ). Exploratory endpoints and adverse events were also analyzed.

**Results:**

Among the 534 subjects, the GSP301 group demonstrated statistically significant improvements in the average rTNSS compared with the OLO group [posterior least square mean difference (LSMD) = −0.56; *P* < 0.0001] and the MF group (posterior LSMD = −0.43; *P* < 0.0001). Consistent benefits were observed across secondary endpoints, including iTNSS, rTOSS, RQLQ, and individual nasal and ocular symptoms (all *P* < 0.05). Additionally, GSP301 reduced the levels of interleukin (IL)-5 and eosinophilic cationic protein (ECP) in nasal secretions. Treatment-emergent adverse events (TEAEs) occurred in 11.2%, 13.5%, and 11.3% of patients in the GSP301, OLO, and MF groups, respectively.

**Conclusion:**

Compared with OLO and MF, GSP301 demonstrated superior efficacy, safety, and potential advantages in alleviating local inflammation in patients with moderate-to-severe SAR.

## Introduction

Allergic rhinitis (AR), a prevalent immunoglobulin E-mediated upper airway disorder, poses a global health challenge. Patients endure substantial disease burdens with significant quality-of-life impairment.^1^ AR affects approximately 10–40% of the worldwide population,^2^ with epidemiological studies in China reporting a self-reported prevalence of 17.6% across 18 major cities and a pollen-induced AR incidence of 18.5%.^3^^,^^4^ Clinically, AR is categorized into seasonal AR (SAR) and perennial AR (PAR) on the basis of allergen exposure patterns, and as “mild” or “moderate/severe” AR on the basis of the effects of symptoms on daily activities.^2^ Classic manifestations include nasal congestion, itching, sneezing, and rhinorrhea, which are often accompanied by ocular symptoms, palate itching, and cough. The prevalence of rhinoconjunctivitis has risen steadily over the past decade,^5^ with 75%–80% of U.S. patients reporting moderate-to-severe symptoms.^6^

The global economic burden of AR has substantially increased over time. In the United States, total annual expenditure rose from $6.1 billion in 2000 to $11.2 billion in 2005.^7^ By 2020, per-patient costs in Dutch healthcare systems reached €4827 annually.^8^ Similarly, Chinese AR expenditures average €195.6 per patient annually, contributing to aggregate societal costs of approximately €440.9 million per year.^9^ Indirect costs further compound this burden, with annual productivity losses in Europe estimated at €30–€50 billion due to work impairment.^10^^,^^11^ Given this substantial economic burden, the development of cost-effective therapeutic strategies that alleviate clinical symptoms while mitigating socioeconomic consequences is clinically imperative.

Current AR management strategies involve 4 principal modalities: allergen avoidance, pharmacotherapy, allergen immunotherapy, and surgical intervention. While allergen minimization is the foundational management approach, it is often impractical for polysensitized patients, as comprehensive environmental controls are clinically unfeasible.^12^ Consequently, pharmacologic interventions emerge as the cornerstone of AR management.

Second-generation H_1_-antihistamines, intranasal corticosteroids (INCSs), and fixed-dose combinations (FDCs) of INCSs with H_1_-antihistamines constitute first-line pharmacotherapy for AR.^13^ Despite the established role of INCS monotherapy, only 60% of patients achieve satisfactory symptom control.^14^ Furthermore, while initial therapeutic onset occurs within 12 h, maximal clinical efficacy requires several days of continuous treatment.^14^^,^^15^ In comparison, INCS-antihistamine FDCs demonstrate superior tolerability compared with their individual components when they are administered separately. These combinations are therefore recommended for patients with suboptimal responses to INCS monotherapy. Bernstein et al demonstrated that INCS-antihistamine combinations achieve therapeutic onset within 5 min,^16^ offering significantly faster relief compared with INCS alone. Currently, 2 FDC formulations are clinically available: MP-Aze (fluticasone with azelastine) and GSP301 (mometasone furoate with olopatadine hydrochloride). Notably, the efficacy of MP-AzeFlu has been clinically validated in Chinese AR populations.^17^

GSP301 is an FDC nasal spray containing the olopatadine hydrochloride (665 μg) and the mometasone furoate (25 μg). Extensive clinical trials in U.S. populations have established its efficacy and safety for the treatment of SAR 18, 19, 20, 21. Its therapeutic benefits also extend to perennial AR and pediatric SAR.^22^^,^^23^ Notably, previous efficacy and safety assessments have been based on comparisons between GSP301 and the sponsor's formulations of olopatadine and mometasone furoate. However, data on the effectiveness and safety profiles of GSP301 specifically in Chinese patients with SAR are lacking.

Therefore, this study aimed to evaluate the efficacy and safety of GSP301 compared with those of its commercially available monocomponents [mometasone furoate (MF) and olopatadine hydrochloride (OLO)] and to investigate local inflammatory changes in Chinese patients with moderate-to-severe SAR.

## Patients and methods

### Ethics

This multicenter study was conducted across 27 centers in mainland China in compliance with Good Clinical Practice and in accordance with the Declaration of Helsinki and the International Conference on Harmonization guidelines. The protocol was reviewed and approved by the Ethics Committee of each study center, and all the legally acceptable representatives (LARs) provided written informed consent. This study is registered on www.chinadrugtrials.org.cn, with registration No. CTR20213407.

### Study design

**Supplementary Fig. 1:**
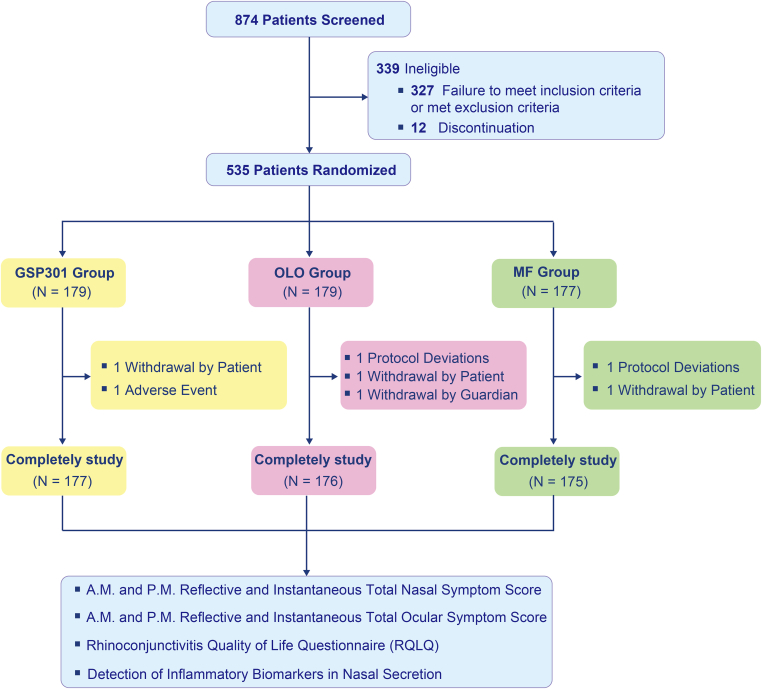
Patient disposition and study design

This was a phase III, double-blind, randomized, parallel-group study conducted in adult and adolescent patients with moderate-to-severe SAR. The study consisted of 2 periods: the placebo run-in period (7–10 days from the screening visit to the randomization visit) and the treatment period (15–17 days from the randomization visit to the final treatment visit) (Fig. S1). After completion of the placebo run-in period, eligible patients were equally randomized to 1 of 3 intranasal treatments for 14 days: GSP301 (665 μg olopatadine hydrochloride and 25 μg of mometasone furoate, 2 sprays/each nostril twice daily), olopatadine hydrochloride (OLO) (Patanase®; 665 μg, 2 sprays/each nostril twice daily), or mometasone furoate (Nasonex®; 50 μg, 1 spray/each nostril twice daily).

### Patients

Eligible patients were ≥12 years old with a clinical history of SAR for ≥2 years before the screening visit and a positive skin-prick test result (with a diameter at least 5 mm greater than that of the negative control) or positive serum-specific IgE (above 0.35 kU/L) for the relevant seasonal allergens. Patients also needed to have a minimum 12-h reflective total nasal symptom score (rTNSS) ≥ 8 points out of 12 points and an A.M. nasal obstruction score ≥2 points out of 3 points at the screening visit.

The key exclusion criteria were acute or chronic rhinosinusitis with/without nasal polyps or other clinical respiratory tract malformations, disorders, or infections; nasal trauma or nasal septal deviation; atopic dermatitis; chronic purulent postnasal drip; rhinitis medicamentosa; active pulmonary disorder or infection (eg, bronchitis, pneumonia, or influenza); upper respiratory tract or sinus infection (within 14 days of the screening visit); or the development of respiratory infections (during the placebo run-in period).

### Assessments

Patients self-assessed reflective and instantaneous nasal symptoms (including nasal obstruction, runny nose, itching, and sneezing) and ocular symptoms (including itching/burning eyes, tearing/watering eyes, and redness of eyes) twice daily (A.M. and P.M.), which were recorded in a symptom diary before self-administering the study medication. Symptoms were rated on a scale from 0 (absent) to 3 (severe) and were reported as reflective (over the previous 12 h) and instantaneous (over the 10 min before dosing). The rTNSS or instantaneous TNSS (iTNSS) was defined as the sum of the 4 nasal-related symptom scores, with a maximum score of 12. The reflective total ocular symptom score (rTOSS) or the instantaneous total ocular symptom score (iTOSS) was defined as the sum of 3 ocular symptom scores with a maximum score of 9. The rhinoconjunctivitis quality-of-life questionnaire (RQLQ) was administered before randomization and at the end of the study. Safety was assessed by monitoring adverse events (AEs), laboratory assessments, and vital signs and conducting electrocardiograms; physical examinations; and ear, nose, and throat examinations.

### End points

The primary efficacy endpoint included the mean changes in average A.M. and P.M. 12-hour rTNSS from baseline to the end of the 14-day treatment, which were compared as follows: GSP301 versus OLO and GSP301 versus MF. The secondary efficacy endpoints included the following: the mean change in average A.M. and P.M. 12-hour iTNSS and rTOSS from baseline to the end of the 14-day treatment and the mean change from baseline to day 15 in the overall RQLQ score, all of which underwent the same between-group comparisons as the rTNSS results did. Additional efficacy end points included the mean change from baseline in the following measurements: average A.M. and P.M. 12-hour rTNSS and iTNSS for each day, average A.M. and P.M. 12-hour reflective and current individual nasal symptoms over the 14-day treatment period, and average A.M. and P.M. 12-hour iTOSS over the 14-day treatment period. The additional efficacy end point results underwent the same comparisons as the rTNSS results did (described above). Laboratory assessments, electrocardiograms, and physical examinations were conducted at the screening and final visits, and ear, nose, and throat examinations and monitoring of vital signs and AEs occurred throughout the study.

### Exploratory end points

We measured the concentrations of local nasal inflammatory biomarkers in nasal secretions at baseline and 14 days after treatment. The concentrations of twenty-five mediators were determined as previously described. The concentrations of eosinophilic cationic protein (ECP) and immunoglobulin E (IgE) were measured using the UniCAP system. Myeloperoxidase (MPO) was measured with a Quantikine Elisa kit (R&D Systems, DMYE00B). The other twenty-two biomarkers, namely, IL-1α, IL-1β, IL-1ra, IL-2, IL-4, IL-5, IL-6, IL-8, IL-10, IL-17, TNF-α, IFN-γ, CCL2, CCL3, CCL4, CCL5, CXCL5, FGF basic, G-CSF, GM-CSF, thrombopoietin (Tpo), and vascular endothelial growth factor (VEGF), were measured using a Luminex Performance assay (R&D Systems, FCSTM03-22).

### Statistical analyses

Efficacy analyses were based on the full analysis set (FAS), defined as all randomized patients who received 1 or more doses of the study drug and completed 1 or more post baseline primary efficacy assessments. Safety assessments were based on the safety analysis set (SAS), which included all randomized patients who received 1 or more doses of the study drug.

Efficacy end points were analyzed via a robust Bayesian approach, which was based on the mixed model for repeated measures (MMRM). The posterior parameters, least square mean difference (LSMD) and its standard error (SE) were used to evaluate the treatment effect. A treatment difference of at least 0.23 units (direct anchor-based regression methodology) on the TNSS was considered clinically meaningful [defined as the minimal clinically important difference (MCID)].^24^ The MCID of the overall RQLQ score was 0.5.^25^ The baseline RQLQ score was recorded on day 1 (visit 2). The statistical significance level was set at *α* = 0.05 (2-sided) for the analyses.

## Results

### Patient disposition and demographics

A total of 535 patients were randomized (1 patient in the OLO group withdrew prior to the administration of the first dose), and 528 patients completed the study Supplementary Fig. 1; 534 were included in the FAS and SAS. All the enrolled patients had a mean (± standard deviation) age of 36.1 (±10.52) years and presented with moderate-to-severe nasal and ocular symptoms. The demographic characteristics, nasal and ocular symptoms and quality of life (QoL) scores were similar between the treatment groups at baseline (Table 1).

**Table 1:** Demographics and baseline assessment scores.

	GSP301 group	OLO group	MF group	Total
No. of subjects	179	178	177	534
Age, mean ± SD	36.0 ± 9.96	35.7 ± 11.37	36.5 ± 10.22	36.1 ± 10.52
Gender				
Male, n (%)	86 (48.0%)	85 (47.8%)	91 (51.4%)	262 (49.1%)
Female, n (%)	93 (52.0%)	93 (52.2%)	86 (48.6%)	272 (50.9%)
Comorbid with asthma, n (%)	4 (2.2%)	9 (5.1%)	3 (1.7%)	16 (3.0%)
Allergen				
Mugwort, n (%)	124 (69.3%)	117 (65.7%)	124 (70.1%)	356 (68.4%)
Ragweed, n (%)	85 (47.5%)	77 (43.3%)	79 (44.6%)	241 (45.1%)
Cypress, n (%)	52 (29.1%)	52 (29.2%)	49 (27.7%)	153 (28.7%)
Birch, n (%)	26 (14.5%)	34 (19.1%)	31 (17.5%)	91 (17.0%)
Humulus, n (%)	60 (33.5%)	56 (31.5%)	51 (28.8%)	167 (31.3%)
Others, n (%)	7 (3.9%)	11 (6.2%)	14 (7.9%)	32 (6.0%)
Average A.M. and P.M. rTNSS, mean ± SD	10.20 ± 1.411	10.13 ± 1.332	10.25 ± 1.431	10.19 ± 1.390
Average A.M. and P.M. iTNSS, mean ± SD	10.12 ± 1.457	10.00 ± 1.431	10.15 ± 1.518	10.09 ± 1.468
Average A.M. and P.M. rTOSS, mean ± SD	7.12 ± 1.321	7.07 ± 1.241	7.22 ± 1.208	7.14 ± 1.257
Average A.M. and P.M. iTOSS, mean ± SD	7.05 ± 1.356	7.01 ± 1.329	7.17 ± 1.317	7.08 ± 1.333
RQLQ score, mean ± SD	4.26 ± 1.223	4.19 ± 1.119	4.26 ± 1.179	4.24 ± 1.173

MF, mometasone furoate. OLO, olopatadine hydrochloride. SD, standard deviation; rTNSS, reflective total nasal symptom score; iTNSS, instantaneous total nasal symptom score; rTOSS, reflective total ocular symptom score; iTOSS, instantaneous total ocular symptom score; RQLQ, rhinoconjunctivitis quality-of-life questionnaire

### Patient-reported nasal symptoms

#### Average A.M. and P.M. rTNSS and iTNSS

**Fig. 1:**
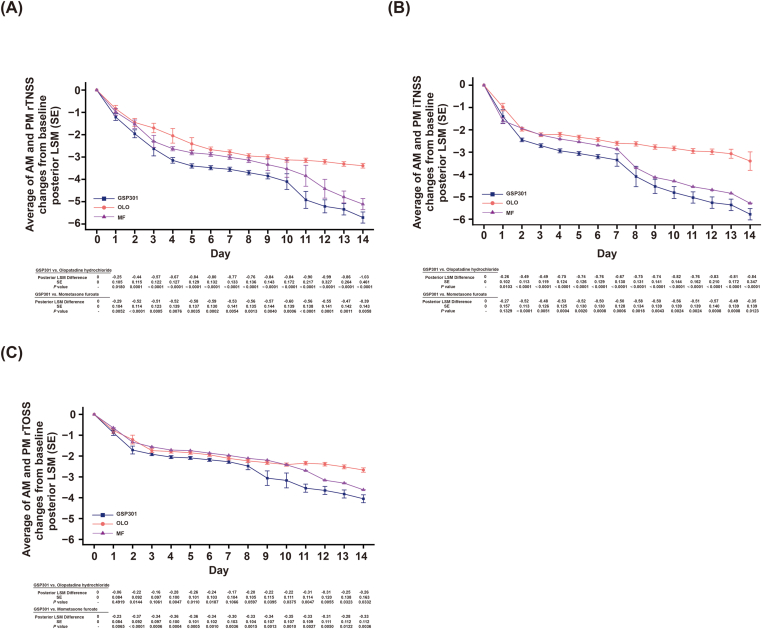
**(A)** Change from baseline in average A.M. and P.M. subject-reported rTNSS for each day. (**B)** Change from baseline in average A.M. and P.M. subject-reported iTNSS for each day. **(C)** Change from baseline in average A.M. and P.M. subject-reported rTOSS for each day. *This figure shows the Bayesian analysis results, where the scatter points represent the posterior LSM in each group and the error bars represent the posterior SE in each group. The corresponding intergroup comparison results (posterior LSM difference, posterior SE and P values) are shown in the table below*

Compared with each individual monotherapy, GSP301 demonstrated statistically significant and clinically meaningful improvements in average A.M. and P.M. 12-hour rTNSS from baseline to the end of the 14-day treatment (all *P* < 0.0001 with GSP301 vs. OLO and GSP301 vs. MF) (Table 2). On individual days, significant improvements in the average A.M. and P.M rTNSS after GSP301 treatment compared with monotherapies were observed from day 1 through day 14 (Fig. 1A), suggesting sustained daily improvements.

**Table 2:** Treatment comparisons of average A.M. and P.M. rTNSSs and iTNSS over 14 days of treatment.

Treatment Group	n1, n2	Posterior LSMD	95% Credible Interval	*P* value
**Average A.M. and P.M. rTNSS**				
GSP 301 vs. OLO	179, 178	−0.56	-1.27, −0.31	<0.0001
GSP 301 vs. MF	179, 177	−0.43	-0.65, −0.22	<0.0001
**Average A.M. and P.M. iTNSS**				
GSP 301 vs. OLO	179, 178	−0.50	-0.75, −0.28	<0.0001
GSP 301 vs. MF	179, 177	−0.44	-0.65, −0.24	0.0006

MF, mometasone furoate. OLO, olopatadine hydrochloride. rTNSS, reflective total nasal symptom score; iTNSS, instantaneous total nasal symptom score; LSMD, least square mean difference

Similar to the rTNSS results, compared with both monotherapies, GSP 301 demonstrated significant and clinically meaningful improvements in average A.M. and P.M. iTNSS from baseline to the end of the 14-day treatment period (*P* < 0.0001 with GSP301 vs. OLO and *P* = 0.0006 with GSP301 vs. MF) (Table 2). When assessed by individual day, significant improvements with GSP301 treatment vs. monotherapy on average A.M. and P.M. iTNSS were demonstrated from day 2 through day 14 (Fig. 1B).

#### Average A.M. and P.M. reflective individual nasal symptoms

Compared with both monotherapies, GSP 301 significantly improved all average A.M. and P.M. reflective individual nasal symptoms during the 14-day treatment period (all *P* < 0.05) (Table 3).

**Table 3:** Treatment comparisons of average A.M. and P.M. reflective nasal symptom scores of individuals over 14 days of treatment.

Treatment Group	n1, n2	Posterior LSMD	95% Credible Interval	*P* value
**Nasal obstruction**				
GSP 301 vs. OLO	179, 178	−0.17	-0.44, −0.09	<0.0001
GSP 301 vs. MF	179, 177	−0.10	-0.16, −0.04	0.0113
**Runny nose**				
GSP 301 vs. OLO	179, 178	−0.14	-0.37, −0.08	<0.0001
GSP 301 vs. MF	179, 177	−0.11	-0.17, −0.06	<0.0001
**Itching**				
GSP 301 vs. OLO	179, 178	−0.12	-0.17, −0.06	<0.0001
GSP 301 vs. MF	179, 177	−0.12	-0.17, −0.06	<0.0001
**Sneezing**				
GSP 301 vs. OLO	179, 178	−0.12	-0.17, −0.06	<0.0001
GSP 301 vs. MF	179, 177	−0.12	-0.17, −0.06	<0.0001

MF, mometasone furoate. OLO, olopatadine hydrochloride. LSMD, least square mean difference

### Patient-reported ocular symptoms

#### Average A.M. and PM rTOSS

Compared with both monotherapies, GSP301 significantly improved the average A.M. and P.M. 12-hour rTOSS from baseline to the end of the 14-day treatment (*P* = 0.0335 vs. OLO; *P* = 0.0004 vs. MF) (Table 4). On individual days, significant improvements on average A.M. and P.M. rTOSS after GSP301 treatment vs. monotherapy were observed during most of the treatment days (Fig. 1C).

**Table 4:** Treatment comparisons of average A.M. and P.M. rTOSS and RQLQ scores.

Treatment Group	n1, n2	Posterior LSMD	95% Credible Interval	*P* value
**Average A.M. and P.M. rTOSS**				
GSP 301 vs. OLO	179, 178	−0.18	-0.35, −0.01	0.0335
GSP 301 vs. MF	179, 177	−0.30	-0.47, −0.13	0.0004
**RQLQ score**				
GSP 301 vs. OLO	179, 178	−0.36	-0.49, −0.22	<0.0001
GSP 301 vs. MF	179, 177	−0.19	-0.32, −0.06	0.0058

MF, mometasone furoate. OLO, olopatadine hydrochloride. rTOSS, reflective total ocular symptom score; RQLQ, rhinoconjunctivitis quality-of-life questionnaire. LSMD, least square mean difference

#### Average A.M. and P.M. reflective individual ocular symptoms

Compared with both monotherapies, GSP301 significantly improved most of the average A.M. and P.M. reflective individual ocular symptoms during the 14-day treatment period (*P* < 0.05 for all comparisons except GSP 301 vs. OLO for redness of the eyes) (Table 5).

**Table 5:** Treatment comparisons of average A.M. and P.M. reflective ocular symptom scores of individuals over 14 days of treatment.

Treatment Group	n1, n2	Posterior LSMD	95% Credible Interval	*P* value
**Itching/Burning eyes**				
GSP 301 vs. OLO	179, 178	−0.06	-0.12, −0.0030	0.0362
GSP 301 vs. MF	179, 177	−0.10	-0.15, −0.04	0.0005
**Tearing/Watering eyes**				
GSP 301 vs. OLO	179, 178	−0.06	-0.12, −0.0030	0.0378
GSP 301 vs. MF	179, 177	−0.10	[-0.16, −0.04]	0.0004
**Redness of eyes**				
GSP 301 vs. OLO	179, 178	−0.06	-0.11, 0.0010	0.0531
GSP 301 vs. MF	179, 177	−0.10	-0.16, −0.04	0.0005

MF, mometasone furoate. OLO, olopatadine hydrochloride. LSMD, least square mean difference

### Rhinoconjunctivitis quality-of-life questionnaire

A significant improvement in the overall RQLQ score from baseline to day 15 was observed with GSP301 vs. both monotherapies (*P* < 0.0001 with GSP301 vs. OLO; *P* = 0.0058 with GSP301 vs. MF) (Table 4).

### Exploratory end points

**Fig. 2:**
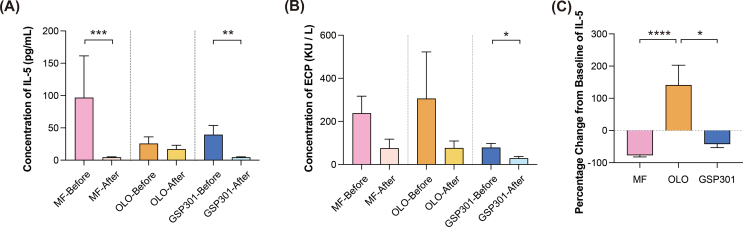
**(A)** Concentrations of IL-5 at baseline and 14 days after the 3 treatments. **(B)** Concentrations of ECP at baseline and 14 days after the 3 treatments. **(C)** The percentage change in IL-5 levels between baseline and 14 days after treatment.

Exploratory testing was performed on 12 patients in the MF group, 12 patients in the OLO group, and 15 patients in the GSP301 group. IL-5 levels significantly decreased from baseline to 14 days in both the MF group and GSP301 group (*P* = 0.0002 and 0.0025, respectively) (Fig. 2A). A reduction in ECP level was exclusively observed in the GSP301 group (*P* = 0.0352) (Fig. 2B). Further comparison of the mean percentage changes from baseline in the IL-5 concentration revealed that GSP301 or MF treatment resulted in significantly greater changes than OLO treatment did (Fig. 2C). However, no differences were observed in any of the other exploratory endpoints between baseline and 14 days or among the 3 groups.

### Safety

The incidence of adverse events was 11.2% with GSP301 treatment, 13.5% with OLO treatment, and 11.3% with MF treatment, the percentage of patients reporting TEAEs was generally similar among treatments (Table 6). All the TEAEs were mild or moderate in severity, and no severe TEAEs were reported. Most of the TEAEs were not considered related to treatment.

**Table 6:** Adverse events occurred during the study.

	GSP 301 (N = 179) N (%)	OLO (N = 178) N (%)	MF (N = 177) N (%)	Total (N = 534) N (%)
Any TEAE	20 (11.2)	24 (13.5)	20 (11.3)	64 (12.0)
TEAE occurring in ≥1% of patients in either group				
High density lipoprotein decreased	3 (1.7)	2 (1.1)	1 (0.6)	6 (1.1)
Eosinophil percentage increased	0	0	4 (2.3)	4 (0.7)
Basophil percentage increased	0	0	3 (1.7)	3 (0.6)
Blood creatine phosphokinase increased	1 (0.6)	0	2 (1.1)	3 (0.6)
Nasal dryness	0	3 (1.7)	0	3 (0.6)
Allergic cough	0	2 (1.1)	0	2 (0.4)
Any TEAE leading to death	0	0	0	0
Any serious TEAE	0	0	0	0

MF, mometasone furoate. OLO, olopatadine hydrochloride. TEAE, treatment-emergent adverse event.

The most frequently reported (occurring in ≥1% of the subjects in any of the 3 treatment groups) TEAEs were decreased high-density lipoprotein levels (n = 6), increased eosinophil percentage (n = 4), increased basophil percentage (n = 3), increased blood creatine phosphokinase levels (n = 3), nasal dryness (n = 3), and allergic cough (n = 2). One subject experienced a TEAE (COVID-19) that led to withdrawal from the study (GSP301 group), and it was considered unrelated to the study treatment and moderate in severity. There was no serious TEAEs occurred in this study.

## Discussion

This multicenter study demonstrated the superior efficacy of GSP301 (mometasone furoate and olopatadine hydrochloride FDC nasal spray) over its individual components, mometasone furoate (MF) and olopatadine hydrochloride (OLO), in Chinese patients (≥12 years) with moderate-to-severe SAR. The primary endpoint analysis revealed statistically and clinically significant improvements in average A.M. and P.M. rTNSS with GSP301, with sustained advantages throughout the 14-day treatment period. Notably, our study used commercially available formulations of MF (Nasonex®) and OLO (Patanase®) rather than a sponsor formulation, enhancing the real-world clinical relevance.

Olopatadine hydrochloride is a selective histamine H1-receptor antagonist that exhibits multimodal antiallergic properties through mast cell stabilization, effectively inhibiting allergen-induced release of histamine, lysozyme, and albumin while also suppressing histamine-driven inflammatory cytokine production in conjunctival mast cells.^16^ Mometasone furoate complements this action through potent local anti-inflammatory effects, preventing the release of allergic mediators and demonstrating a clinically meaningful onset of action of 12 h.^16^^,^^26^ Clinical evidence has demonstrated that olopatadine hydrochloride nasal spray significantly improves the TNSS, ocular symptoms and RQLQ scores,^27^ whereas mometasone furoate nasal spray also provides effective control of moderate-to-severe SAR symptoms, including ocular manifestations.^28^ The GSP301 capitalizes on this mechanistic synergy, with each component acting through distinct but complementary pathways to enhance overall allergic rhinitis symptom control. Importantly, compared with either monotherapy, GSP301 has favorable bioavailability and tolerability profiles,^19^ making it a valuable therapeutic option for comprehensive SAR management.

The amelioration of ocular symptoms, including itching, tearing and redness, constitutes a critical quality-of-life consideration for SAR patients with concurrent allergic conjunctivitis. Our comparative analysis revealed that compared with either MF or OLO monotherapy, GSP301 provided superior ocular symptom relief, with significantly greater improvements in average A.M. and P.M. rTOSS. Notably, a meta-analysis indicated only modest TOSS improvement with MF treatment versus placebo (SMD = −0.29),^29^ whereas GSP301 demonstrated clinically meaningful ocular benefits throughout the 14-day treatment period. Importantly, this nasal spray formulation provided simultaneous nasal and ocular symptom control, representing a distinct advantage over oral antihistamines or ophthalmic solutions that exclusively target ocular symptoms. Temporal analysis confirmed the consistent therapeutic superiority of GSP301 over MF across all assessment timepoints, with an advantage over OLO on 71% of treatment days (10 out of 14).

The RQLQ comprehensively evaluates nasal and ocular symptom severity and quality-of-life impact in SAR patients. After 14 days of treatment, all groups demonstrated improved RQLQ scores; however, the GSP301 group achieved significantly greater improvement than both the MF and OLO monotherapy groups did. In addition to significantly improving the TNSS, RQLQ outcomes provided equally robust evidence to support clinical medication selection. In addition, GSP301 demonstrated superior efficacy in enhancing overall quality of life. These results align with and extend previous clinical evidence demonstrating GSP301's superiority over sponsor-formulated mometasone furoate and olopatadine hydrochloride monotherapies.

Group 2 innate lymphoid cells (ILC2s) are critical effectors of type 2 immunity in airway inflammation, establishing an IL-5-dominated inflammatory microenvironment associated with type 2 cytokines.^30^ Building on this mechanistic foundation, our study demonstrated that GSP301 significantly reduces local concentrations of both IL-5 and ECP, with the latter being a well-established biomarker of eosinophil inflammation and a diagnostic indicator for AR.^31^ Whereas MF also effectively reduced the expression of IL-5, GSP301 significantly suppressed the expression of type 2 cytokines, providing a mechanistic foundation for its superior TNSS outcome.

Throughout the 14-day treatment period, the incidence of TEAE with GSP301 was comparable to that with MF and lower than that with OLO. Nasal bleeding occurred in 3 patients in total, with 1 case reported in each treatment group. Overall, GSP301 demonstrated a favorable safety profile with excellent tolerability. Most TEAEs had no or minor physiological functional impact. As previous SAR clinical trials have confirmed the superiority of GSP301 over placebo in rTNSS and iTNSS improvement, we did not compare the safety and effectiveness compared with placebo in the present study.^18^^,^^20^

This clinical trial provides clinically actionable guidance by demonstrating the superior efficacy of GSP301 compared with that of real-word commercially available mometasone furoate (Nasonex®) and olopatadine hydrochloride (Patanase®). Bayesian statistical methods further reinforced the robustness of our findings. Our exploratory analysis revealed that GSP301 modulates local type 2 inflammation in SAR patients. Although clinical observations are limited to short-term outcomes, this anti-inflammatory effect may confer long-term therapeutic benefits. This study has several limitations. First, the patient follow-up period was only 14 days, precluding the assessment of long-term efficacy. Second, the number of patients for the exploratory endpoints was relatively small. Future studies should involve larger sample sizes and incorporate multidimensional research approaches.

In conclusion, treatment selection for SAR should be guided by symptom severity and individualized therapeutic goals, with clinicians prioritizing pharmacotherapies that optimize cost-efficacy profiles while advancing precision care principles. Compared with MF or OLO monotherapy, GSP301 demonstrated efficacy and favorable tolerability, achieving statistically significant and clinically meaningful improvements in moderate-to-severe SAR patients.

## Abbreviations

AR, allergic rhinitis; SAR, seasonal allergic rhinitis; PAR, perennial allergic rhinitis; OLO, olopatadine hydrochloride; MF, mometasone furoate; TNSS, total nasal symptom score; rTNSS, reflective total nasal symptom score; iTNSS, instantaneous total nasal symptom score; iTOSS, instantaneous total ocular symptom score; RQLQ, rhinoconjunctivitis quality-of-life questionnaire; AE, adverse event; LSM, least square mean; ECP, eosinophilic cationic protein; INCS, intranasal corticosteroids; FDC, fixed dose combination; LAR, legally acceptable representative; IgE, immunoglobin E; MPO, myeloperoxidase; IL, interleukin; TNF-α, tumor necrosis factor α; IFN-γ, interferon γ; CCL, C-C motif chemokine ligand; CXCL, C-X-C motif chemokine ligand; FGF, fibroblast growth factor; G-CSF, granulocyte colony stimulating factor; GM-CSF, granulocyte macrophage colony stimulating factor; Tpo, thrombopoietin; VEGF, vascular endothelial growth factor; FAS, full analysis set; SAS, safety analysis set; MMRM, mixed model for repeated measures; SE, standard error; MCID, minimal clinically important difference; TEAE, treatment emergent adverse event; SAE, serious adverse event; SMD, standardized mean difference; ILC2s, group 2 innate lymphoid cells.

## Disclosure statement

Nothing to disclose.

## Availability of data and material

The data are available from the corresponding author upon reasonable request.

## Consent for publication

All authors have read the final manuscript and agreed to publication of the work.

## Ethics approval

This study was approved by the Ethics Committee of Beijing Tongren Hospital and Chinese Clinical Trial Registry. And written informed consent was obtained from all participants. This study is registered on www.chinadrugtrials.org.cn, with registration No. CTR20213407.

## Author contribution

YTSM, XYW, XDW, and LZ conceived and designed the study; TZ, ZWC, WC, FQ, XYR, YY, SPB, LFX, CQZ, QNZ, ZMX, HFZ, JJC, QQH, LZ, XBZ, XC, CL, RXM, HZ, ZDX, and MH were responsible for the management of clinical trial, and data collection; YTSM, XYW, XDW, and LZ wrote the draft and revised manuscript; all authors approved the final version of this manuscript.

## Funding

This study was funded by Grand Pharmaceutical Group Ltd. (Beijing Grand Johamu Pharmaceutical Company,Ltd.) and conducted in accordance with Good Publication Practice (GPP) guidelines.

## Declaration of competing interest

All authors declare no financial or commercial conflicts of interest.

## References

[1] Meltzer E.O. (2016). Allergic rhinitis: burden of illness, quality of life, comorbidities, and control. Immunology and Allergy Clinics.

[2] Bousquet J., Khaltaev N., Cruz A.A. (2008). Allergic rhinitis and its impact on asthma (ARIA) 2008 update (in collaboration with the world health organization, GA(2)LEN and AllerGen). Allergy.

[3] Wang X.Y., Ma T.T., Wang X.Y. (2018). Prevalence of pollen-induced allergic rhinitis with high pollen exposure in grasslands of northern China. Allergy.

[4] Wang X.D., Zheng M., Lou H.F. (2016). An increased prevalence of self-reported allergic rhinitis in major Chinese cities from 2005 to 2011. Allergy.

[5] Sasaki M., Morikawa E., Yoshida K., Adachi Y., Odajima H., Akasawa A. (2019). The change in the prevalence of wheeze, eczema and rhino-conjunctivitis among Japanese children: findings from 3 nationwide cross-sectional surveys between 2005 and 2015. Allergy.

[6] Meltzer E.O., Farrar J.R., Sennett C. (2017). Findings from an online survey assessing the burden and management of seasonal allergic rhinoconjunctivitis in US patients. J Allergy Clin Immunol Pract.

[7] Blaiss M.S. (2010). Allergic rhinitis: direct and indirect costs. Allergy Asthma Proc.

[8] Avdeeva K.S., Reitsma S., Fokkens W.J. (2020). Direct and indirect costs of allergic and non-allergic rhinitis in the Netherlands. Allergy.

[9] Li X., Xu X., Li J. (2022). Direct and indirect costs of allergic and non-allergic rhinitis to adults in Beijing, China. Clin Transl Allergy.

[10] Vandenplas O., Vinnikov D., Blanc P.D. (2018). Impact of rhinitis on work productivity: a systematic review. J Allergy Clin Immunol Pract.

[11] Zuberbier T., Lötvall J., Simoens S., Subramanian S.V., Church M.K. (2014). Economic burden of inadequate management of allergic diseases in the European Union: a GA(2) LEN review. Allergy.

[12] Wise S.K., Lin S.Y., Toskala E. (2018). International consensus statement on allergy and rhinology: allergic rhinitis. Int Forum Allergy Rhinol.

[13] Bousquet J., Anto J.M., Bachert C. (2020). Allergic rhinitis. Nat Rev Dis Primers.

[14] Kumar R., Jain M.K., Kushwaha J.S. (2022). Efficacy and safety of fluticasone furoate and oxymetazoline nasal spray: a novel first fixed dose combination for the management of allergic rhinitis with nasal congestion. J Asthma Allergy.

[15] Baroody F.M., Brown D., Gavanescu L., DeTineo M., Naclerio R.M. (2011). Oxymetazoline adds to the effectiveness of fluticasone furoate in the treatment of perennial allergic rhinitis. J Allergy Clin Immunol.

[16] Bernstein J.A., Bernstein J.S., Makol R., Ward S. (2024). Allergic rhinitis: a review. JAMA.

[17] Zhou B., Cheng L., Pan J. (2023). A clinical study to assess the efficacy and safety of MP-AzeFlu nasal spray in comparison to commercially available azelastine hydrochloride and fluticasone propionate nasal sprays in Chinese volunteers with allergic rhinitis. Pulm Ther.

[18] Gross G.N., Berman G., Amar N.J., Caracta C.F., Tantry S.K. (2019). Efficacy and safety of olopatadine-mometasone combination nasal spray for the treatment of seasonal allergic rhinitis. Ann Allergy Asthma Immunol.

[19] Patel P., Salapatek A.M., Tantry S.K. (2019). Effect of olopatadine-mometasone combination nasal spray on seasonal allergic rhinitis symptoms in an environmental exposure chamber study. Ann Allergy Asthma Immunol.

[20] Hampel F.C., Pedinoff A.J., Jacobs R.L., Caracta C.F., Tantry S.K. (2019). Olopatadine-mometasone combination nasal spray: evaluation of efficacy and safety in patients with seasonal allergic rhinitis. Allergy Asthma Proc.

[21] Andrews C.P., Mohar D., Salhi Y., Tantry S.K. (2020). Efficacy and safety of twice-daily and once-daily olopatadine-mometasone combination nasal spray for seasonal allergic rhinitis. Ann Allergy Asthma Immunol.

[22] Prenner B.M., Amar N.J., Hampel F.C., Caracta C.F., Wu W. (2022). Efficacy and safety of GSP301 nasal spray in children aged 6 to 11 years with seasonal allergic rhinitis. Ann Allergy Asthma Immunol.

[23] Segall N., Prenner B., Lumry W., Caracta C.F., Tantry S.K. (2019). Long-term safety and efficacy of olopatadine-mometasone combination nasal spray in patients with perennial allergic rhinitis. Allergy Asthma Proc.

[24] Barnes M., Vaidyanathan S., Williamson P., Lipworth B. (2010). The minimal clinically important difference in allergic rhinitis. Clin Exp Allergy.

[25] Juniper E.F., Guyatt G.H., Griffith L.E., Ferrie P.J. (1996). Interpretation of rhinoconjunctivitis quality of life questionnaire data. J Allergy Clin Immunol.

[26] Ridolo E., Barone A., Nicoletta F. (2023). Intranasal corticosteroid and antihistamine combinations in the treatment of allergic rhinitis: the role of the novel formulation olopatadine/mometasone furoate. Expet Rev Clin Immunol.

[27] Meltzer E.O., Hampel F.C., Ratner P.H. (2005). Safety and efficacy of olopatadine hydrochloride nasal spray for the treatment of seasonal allergic rhinitis. Ann Allergy Asthma Immunol.

[28] Hebert J.R., Nolop K., Lutsky B.N. (1996). Once-daily mometasone furoate aqueous nasal spray (Nasonex) in seasonal allergic rhinitis: an active- and placebo-controlled study. Allergy.

[29] Soe K.K., Krikeerati T., Pheerapanyawaranun C., Niyomnaitham S., Phinyo P., Thongngarm T. (2023). Comparative efficacy and acceptability of licensed dose intranasal corticosteroids for moderate-to-severe allergic rhinitis: a systematic review and network meta-analysis. Front Pharmacol.

[30] Peng Y.-Q., Qin Z.-L., Fang S.-B. (2020). Effects of myeloid and plasmacytoid dendritic cells on ILC2s in patients with allergic rhinitis. J Allergy Clin Immunol.

[31] Xi Y., Deng Y.-Q., Li H.-D. (2022). Diagnostic value of a novel eosinophil cationic protein-myeloperoxidase test paper before and after treatment for allergic rhinitis. J Asthma Allergy.

